# Sirtuins, aging and diseases

**DOI:** 10.1186/1753-6561-6-S3-O8

**Published:** 2012-06-01

**Authors:** Leonard Guarente

**Affiliations:** 1 Paul F. Glenn Lab and Department of Biology, MIT, Cambridge, MA 02139, USA

## 

SIR2 and related genes (sirtuins) are NAD-dependent deacetylases that link metabolism, protein acetylation and aging in a variety of species. Sirtuins are also involved in the longevity conferred by dietary or calorie restriction (CR). The mammalian sirtuins SIRT1-7 are involved in changes in stress resistance and metabolism that are triggered by CR, which not only extends life span, but also protects against many diseases of aging. In this talk, I will describe how mamamlian SIRT1 impacts tissue metabolism and diseases by deacetylating nuclear transcription factors that govern key physiological pathways. I will also describe new data from several labs showing that the mitochondrial sirtuin SIRT3 suppresses reactive oxygen species (ROS) in mitochondria and thus links sirtuins, calorie restriction, ROS, mitochondria and aging. The effect of SIRT3 on mitochondrial generated ROS further regulates the key nuclear transcription factor HIF-1α, and may thus govern the metabolic reprogramming (Warburg effect) in cancer. Finally, I will touch upon recent progress in trying to understand the mechanism of action of SIRT1-activating compounds, which are currently in numerous human clinical trials.

Supported by NIA & Glenn Foundation for Medical Research. LG consults for Sirtris/GSK.

